# Micronutrient Deficiencies in Laparoscopic Sleeve Gastrectomy

**DOI:** 10.3390/nu12092896

**Published:** 2020-09-22

**Authors:** Omar Jamil, Raquel Gonzalez-Heredia, Pablo Quadri, Chandra Hassan, Mario Masrur, Reed Berger, Karen Bernstein, Lisa Sanchez-Johnsen

**Affiliations:** 1 Department of Internal Medicine, University of Chicago, Chicago, IL 60637, USA; Omar.Jamil@uchospitals.edu; 2 Department of Surgery, University of Illinois at Mount Sinai Hospital, Chicago, IL 60609, USA; r.gheredia82@gmail.com; 3 Department of Surgery, Saint Louis University, St. Louis, MO 63104, USA; pablo.quadri@health.slu.edu; 4 Department of Surgery, Division of General, Minimally Invasive & Robotic Surgery, University of Illinois at Chicago, Chicago, IL 60612, USA; chandrar@uic.edu (C.H.); mmasrur@uic.edu (M.M.); 5 Departments of Surgery and Medicine, University of Illinois at Chicago, Chicago, IL 60612, USA; rberger@uic.edu; 6 Department of Pediatrics, Division of Adolescent Medicine, University of Illinois at Chicago, Chicago, IL 60612, USA; kbernste@uic.edu; 7 Department of Family Medicine, Rush University Medical Center, Chicago, IL 60612, USA

**Keywords:** laparoscopic sleeve gastrectomy, micronutrients, deficiency

## Abstract

The purpose of this study was to conduct a literature review to examine micronutrient deficiencies in laparoscopic sleeve gastrectomy. We conducted a literature review using PubMed and Cochrane databases to examine micronutrient deficiencies in SG patients in order to identify trends and find consistency in recommendations. Seventeen articles were identified that met the defined criteria. Iron, vitamin B12 and vitamin D were the primary micronutrients evaluated. Results demonstrate the need for consistent iron and B12 supplementation, in addition to a multivitamin, while vitamin D supplementation may not be necessary. Additional prospective studies to establish a clearer picture of micronutrient deficiencies post-SG are needed.

## 1. Introduction

Sleeve Gastrectomy (SG) represented the most frequently performed bariatric procedure in the U.S. in 2017, with rates of bariatric surgery as follows: 59.4% SG, 17.8% Roux-en Y Gastric Bypass (RYGB), 2.8% Laparoscopic Adjustable Gastric Banding (LAGB) and 0.7% Biliopancreatic Diversion with Duodenal Switch (BPD-DS) [1]. SG was previously considered a purely restrictive procedure which was used as a first stage surgery before BPD-DS [2]. A study from 2015 demonstrated that SG resulted in the same level of micronutrient deficiency as gastric banding, a purely restrictive procedure [3]. Since becoming a standalone procedure, SG has quickly become a preferred treatment option. In addition to being a restrictive procedure, sleeve gastrectomy is hypothesized to reduce the hormone ghrelin, which increases hunger [4]. SG is now considered a safe procedure that results in substantial and sustained weight loss, reduction of comorbidities and improvements in quality of life [4]. Moreover, the risk associated with SG is considered to be lower than RYGB [5].

In SG, a gastric tube (sleeve) between 50 and 200 cc in volume is created while the remainder of the stomach is excised [6]. SG has the advantage over other operations of preserving the pylorus of the stomach, thereby avoiding dumping syndrome [7,8]. By eluding the intestinal bypass of the DS and RYGB, many complications are avoided, resulting in a lower mortality rate [8,9,10]. The procedure is safer than other complicated malabsorptive surgeries such as RYGB and BPD-DS and more effective than purely restrictive procedures such as LAGB [11]. Early studies suggested that, unlike RYGB, SG presented no risk for micronutrient deficiency [8,9,12]. More recently, many studies have noted measurable postoperative deficiencies in vitamin B12, vitamin D, iron and folate [9,13,14,15,16,17,18,19,20]. However, disagreements exist about the extent of the postoperative micronutrient deficiencies, the benefits and timing of supplementation and their correlation with preoperative health. Prior to surgery, patients with obesity have a higher incidence of micronutrient deficiencies than the population at large due to the reduced bioavailability of nutrients associated with obesity [21].

Several factors contribute to micronutrient deficiencies in patients who have undergone SG: the reduction in stomach size which results in decreased food intake, a decrease in ghrelin and other gastrointestinal (GI) hormones that reduce appetite, a decreased tolerance of some foods and a reduced metabolism of certain micronutrients, in part due to the loss of intrinsic factor [22]. Moreover, the recommendations for supplementation for SG patients that currently exist are not consistent and often based on data from patients who underwent RYGB and are not unique to SG patients [23]. As such, the purpose of this paper is to review and analyze the findings on micronutrient deficiencies in patients undergoing SG, as a comprehensive review of micronutrient deficiencies for this procedure is lacking.

## 2. Materials and Methods

A literature review was conducted to examine micronutrient deficiencies in SG patients in order to identify trends and determine consistency in recommendations. PubMed and Cochrane database searches were conducted from January 2009 to March 2020 to locate the relevant literature. The key words used were the following: “Sleeve Gastrectomy”, “Micronutrient Deficiency”, “Vitamin D deficiency”, “Vitamin B12 deficiency”, “Iron deficiency” “Bariatric” and “Deficiencies”. Studies that explicitly focused on micronutrient deficiencies while examining a cohort of patients were selected. Studies often included data from patients undergoing RYGB, but only the data from SG patients were extracted and included in the results. Review articles were excluded so that primary studies from review articles could be examined. All values were expressed as mean (standard deviation). All studies in the review cited below prescribed a daily multivitamin postoperatively. Specific postoperative supplementation is described in each relevant section below.

A deficiency was defined as a serum plasma level measured below the reference range for normal. Excess was defined as a serum plasma level above the reference range for normal. Normal levels were defined based on the American Society for Metabolic and Bariatric Surgery Integrated Health Nutritional Guidelines for the Surgical Weight Loss Patient 2016 Update: Micronutrients [24]. Normal limits were as follows: iron (μg/dL) 60–170, ferritin (ng/mL) 12–300 (male) and 12–150 (female), Hb (g/dL) 12–17, B12 (pg/mL) 200–1000, folate (ng/mL) 2.5–20 and vitamin D (ng/mL) 25–65. There was some variation in the definition of the “normal” limits between studies; in some cases, these are either described or discussed within the tables. The percent of patients with deficiencies were described based on the parameters within each respective study.

## 3. Results

As shown in Table 1, 17 studies examined micronutrient deficiencies in SG patients. Sixteen out of the 17 studies were conducted outside of the U.S., with approximately half (*n* = 9) being prospective and half being retrospective (*n* = 8). The mean number of patients included in studies was 84.9 ranging from 30 to 336 patients.

### 3.1. Iron and Ferritin

As shown in Table 2, individuals with obesity are frequently noted to be iron deficient prior to bariatric surgery [9,11] Obesity can result in both an anemia of chronic disease and dysregulation of iron uptake at the level of the enterocytes [32]. Studies have also found that iron deficiency anemia is most likely due to a lack of gastric hydrochloric acid production and the inability to convert iron to a more absorbable form postoperatively [19,23]. The presence of a postoperative iron deficiency could be partially explained by a preoperative deficiency. Eleven of the 17 studies specifically examined iron levels, and, of those, five studies revealed deficiencies in iron levels over time [9,11,14,15,17]. The majority of the studies reviewed did not describe additional iron supplementation, although four studies specifically mentioned supplementation was provided beyond a multivitamin with iron [11,16,17,29]. In the absence of iron supplementation, the operation itself can result in new deficiencies, by either exacerbating those of deficient patients or creating new deficiencies in patients who did not previously have them [33]. These deficiencies were not seen in later studies due to the benefit of iron supplementation in those studies.

As shown in Table 2, the three studies that have the largest numbers of patients with iron deficiencies do not describe a targeted approach to iron supplementation beyond multivitamins [14,15,26]. There was no evidence to suggest that any of these studies had a larger number of women of childbearing age who were menstruating (a group more likely to be iron deficient due to menstruation) versus post-menopausal women, as these data were not reported. The supplementation regimens for these studies are further described in Appendix A [14,15,26]. Out of the three studies that found the fewer patients with iron deficiency post-surgery [16,17,18], two clearly describe an iron supplementation regimen, either with 160 mg of iron with 100% compliance or with iron supplementation in addition to the multivitamin regimen described in other studies [16,17]. Notably, studies that demonstrate a dedicated iron supplementation regimen and demonstrate lower levels of iron deficiency come in later years after the American Society for Metabolic & Bariatric Surgery in 2013 recommended iron supplementation for all bariatric patients [34].

Serum ferritin is an acute phase reactant that is a marker of decreased iron storage and iron deficiency. Table 3 demonstrates that those studies which reported on serum ferritin found declining serum ferritin levels post-surgery, and a number of studies reported that patients had ferritin deficiencies [13,16,19,20,26,30]. An exception to this is Zarshenas and colleagues’ study [20], which was also the only study that did not reveal an iron deficiency. When assessed, ferritin deficiency is much more apparent in SG patients than iron deficiency. This may suggest that, even with normal iron levels, these patients are still iron deficient, as serum ferritin gives a more accurate assessment of the body’s total iron storage [35].

### 3.2. B12 and Folate

Fourteen studies examined B12 levels (Table 4) and demonstrated notable deficiencies in post-bariatric surgery patients that increased over time. Folate, an important DNA precursor, was examined in 13 studies (Table 5). Several studies treated patients with in-office intramuscular B12 injections postoperatively and dosages ranged 1000–3000 mcg. These studies [16,26,29] had a lower rate of patients with B12 deficiencies compared to the other studies without a comparable supplementation regimen. In-office intramuscular injection appeared to eliminate issues related to adherence to vitamin regimens. Moreover, those with poor adherence to a supplementation regimen demonstrated higher levels of vitamin B12 deficiencies at 12 months postoperatively [19]. Patients who received aggressive folic acid regimens had fewer vitamin B12 deficiencies over time [30]. Appendix A also lists additional notes from the studies that were reviewed.

### 3.3. Vitamin D and Calcium

As seen in Table 6, almost all of the studies report a decrease in the proportion of patients with vitamin D deficiencies postoperatively as early as six months postoperatively, sustained until 24 months [11,14,25]. Results from studies with a longer follow up period reveal an increased number of patients with vitamin D deficiencies at 24- and 36-month follow-up [16,17]. It is not clear why more two- and three-year follow-ups demonstrated an increase in deficiencies, but patients with SG often begin regaining weight at the 24-month mark [36]. Notably, the three studies with the largest declines in the number of patients with vitamin D deficiencies postoperatively did not show dedicated vitamin D supplementation regimens beyond a multivitamin postoperatively [18,19,29]. For example, in one intervention, Lanzarini and colleagues provided 16,000 IU calcifediol to patients with preoperative vitamin D deficiencies and no additional supplementation for patients without preoperative deficiencies [31]. Their results reveal that, by 24-month postoperative follow-up, the intervention group (16,000 IU calcifediol for deficient patients) and non-intervention group (baseline multivitamin supplementation for all patients) had similar levels of vitamin D deficiency. In other words, as long as patients did not have a vitamin D deficiency preoperatively, additional vitamin D supplementation was not necessary to achieve satisfactory results. Notably, this is in contrast to RYGB patients where the intervention group benefitted significantly [31]. However, in a study where appropriate supplementation was given, fewer patients were deficient at four-year follow-up in both SG and RYGB groups [37]. Appendix A also lists additional notes regarding supplementation from the studies that were reviewed.

## 4. Discussion

The purpose of this study was to conduct a comprehensive review of the literature on micronutrient deficiencies in post-SG patients. Past reviews have focused on other bariatric procedures, and, to our knowledge, this is the first review to focus exclusively on SG [38]. In terms of iron supplementation, based on the results from these 17 studies, it appears that post-bariatric surgery supplementation with iron beyond a multivitamin resulted in the fewest deficiencies [9,18,20]. This is due to the prevalence of postoperative proton pump inhibitor (PPI) use and a reduction in hydrochloric acid production after the SG, which prevents conversion of the iron supplement to the absorbable ferrous form [14,23]. Iron supplementation can be optimized when taken with vitamin C or citrus fruit to allow the conversion of iron to its most absorbable form [39]. Postoperatively, patients have decreased inflammatory markers and a hypothesized upregulation of iron uptake in the small bowel [40]. As such, based on the studies that were reviewed [11,16,30], it appears that supplementation with iron will aid in decreasing iron deficiencies post-SG.

Vitamin B12 deficiencies are expected post-SG due to the resection of the fundus and the loss of intrinsic factor produced by parietal cells, which are essential in the absorption of B12 [11,14,15,40]. Vitamin B12 and folic acid both need gastric acid to be properly released from food [14]. Gastric acid is reduced by means of the gastrectomy and also proton pump inhibitors use postoperatively. While evidence to suggest a link between bariatric surgery and adverse neonatal outcomes is weak and inconclusive, women of childbearing age should optimize folate levels, as deficiencies may be related to neural tube defects [9,15,41]. Folate deficiencies are possibly caused by food choices rather than a mechanism related to surgery [14,42]. Folate stores are depleted within months unlike vitamin B12, which can be stored in the liver for 1–2 years [14,15,19]. As such, folate deficiencies are likely to appear earlier than B12 deficiencies due to the difference in the body’s storage capacity. Studies with aggressive B12 and folic acid regimens postoperatively, as well as the use of large dose intra-muscular doses for vitamin B12 deficiencies demonstrate the most beneficial outcomes for patients [9,16,26]. Intramuscular dosing of B12 allows direct uptake into the blood stream and avoids the problems of absorption created by post-SG anatomy. Additionally, when deficiencies were corrected preoperatively, there were lower rates of deficiency postoperatively [18].

Patients with obesity may suffer from vitamin D deficiency due to the sequestration of vitamin D in the fat, decreased sun exposure, sedentary lifestyle and the psychological component of covering more skin [18,20,28,43]. Sufficient sunlight is important for adequate levels of vitamin D, and patients with more adipose tissue are less likely to engage in activities such as sunbathing where skin is exposed [44]. Vitamin D is a fat-soluble vitamin that requires lipids absorption for proper uptake into the body and poor lipid absorption can result in vitamin D deficiencies. The vitamin D deficiencies found in postoperative patients may also explain secondary hyperparathyroidism, which was found in 39% of patients [14,15]. Vitamin D is negatively correlated with body fat, as patients with obesity have difficulty metabolizing and storing this nutrient properly [45]. As such, many of these issues seem to resolve with postoperative weight loss. Overall, additional research regarding the benefits of vitamin D supplementation is warranted to determine whether aggressive vitamin D supplementation beyond a multivitamin is indicated in SG patients. Studies that demonstrated very high rates of vitamin D deficiency postoperatively also had high rates preoperatively, and studies in which vitamin D deficiencies were corrected through supplementation preoperatively did not show high rates of deficiency postoperatively [18]. Since vitamin D levels can vary based on skin tone and environmental sunlight exposure [46], it is essential that health care providers are aware of patients’ race/ethnic background and geographic location when making vitamin D supplementation recommendations. Overall, the majority of the studies found vitamin D deficiencies did not develop de-novo postoperatively and improved with supplementation [15,25,31,37]. Vitamin D has been observed as the vitamin most often deficient prior to surgery in patients with obesity [47].

As demonstrated in this review, there is a dearth of research examining micronutrient deficiencies post-SG. Moreover, the efficacy of multivitamin supplementation post-SG, although nearly universally utilized, seems to be poor in short-term follow up [48]. This may be partially due to poor patient adherence in the long term [49], but lack of specific and targeted interventions appear to also play a role. Overall, results from this review indicate that there is a need for prospective, longitudinal studies to better understand how various interventions impact postoperative deficiencies. As both vitamin B12 and vitamin D deficiencies decrease when corrected preoperatively, clear guidelines for preoperative supplementation and prospective studies to affect their efficacy should be explored.

For vitamin B12 supplementation, intramuscular injections should be examined. Such in-office injections reduce issues related to adherence to vitamin regimens and avoid the need for intrinsic factor that is lost during bariatric surgery. Related to this is that there is a need to develop guidelines related to decision-making about the feasibility and efficacy of in-office versus at-home supplementation for vitamin B12 and folate. Results from the studies that were reviewed reveal that vitamin D supplementation regimens beyond a multivitamin postoperatively were not needed to reduce postoperative deficiencies [18,19,29]. As such, additional studies examining the need for aggressive vitamin D supplementation postoperatively should be conducted. In a recent study, a specialized vitamin resulted in fewer de-novo micronutrient deficiencies in long-term follow-up than a more basic multivitamin, and recommendations about the type of specialized multivitamin that is best suited for patients who undergo SG should be explored [50]. Overall, there is a need to conduct additional research studies regarding micronutrient supplementation post-SG in order to develop and augment current guidelines about the timing, dosage and type of supplementation recommended.

Limitations of this review include the fact that the majority of these studies were conducted outside of the U.S. and that many of them had a short follow-up period. In addition, there were variations between the studies in the study design, vitamin supplementation and location that, at times, made it difficult to compare outcomes across studies.

## 5. Conclusions

Current micronutrient supplementation guidelines published by the British Obesity and Metabolic Surgery Society and the American Society for Metabolic & Bariatric Surgery in 2020 and 2019, respectively, will be augmented by this comprehensive review of micronutrient deficiencies in SG patients [51,52]. Between these sets of guidelines, only the latter focuses on recommendations specifically for SG patients and notes weakness in evidence of vitamin recommendations for these patients when compared to other procedures. We hope this review can help support future revisions of guidelines and highlight the need for prospective studies. In particular, we hope that prospective studies can be conducted within the U.S. to appropriately address this patient population. This is critically important because utilizing data from other countries with regards to micronutrient deficiencies may lead to inaccurate recommendations due to the tremendous variation in diet and eating habits between countries, as well as regions of the world with different environments that may also affect micronutrient intake and absorption. 

## Figures and Tables

**Table 1:** Articles reviewed.

Article Number	Year	Authors	Reference #	Prospective vs. Retrospective	Country	# Patients
1	2009	Hakeam et al.	[9]	Prospective	Saudi Arabia	61
2	2010	Gehrer et al.	[11]	Prospective	Switzerland	50
3	2010	Aarts et al.	[14]	Prospective	Netherlands	60
4	2011	Ruiz-Tovar et al.	[25]	Retrospective	Spain	30
5	2011	Kehagias et al.	[26]	Prospective	Greece	30
6	2012	Damms-Machado et al.	[15]	Prospective	Germany	54
7	2012	Moize et al.	[16]	Prospective	Spain	61
8	2012	Saif et al.	[17]	Retrospective	USA	35
9	2012	Capoccia et al.	[27]	Prospective	Italy	138
10	2013	Eltweri et al.	[13]	Retrospective	UK	41
11	2013	Gjessing et al.	[28]	Retrospective	Norway	125
12	2015	Belfiore et al.	[29]	Retrospective	Italy	47
13	2015	Ben-Porat et al.	[30]	Retrospective	Israel	77
14	2015	Lanzarini al.	[31]	Prospective	Spain	96
15	2016	Al-Mulhim	[18]	Prospective	Saudi Arabia	112
16	2016	Gillon et al.	[19]	Retrospective	Norway	336
17	2016	Zarshenas et al.	[20]	Retrospective	Australia	91

**Table 2:** Iron (%): Percentage (%) of patients with deficiencies at each postoperative visit.

Study	Preop (%)	3 Months (%)	6 Months (%)	12 Months (%)	24 Months (%)	36 Months (%)
Hakeam et al.	0		4.9	4.9		
Gehrer et al.	3	2	12	16	18	
Aarts et al.				43		
Kehagias et al.	20					17.8
Damms-Machado et al.	29	39.3	37.9			
Moize et al.	30.8		4.3	10.3	9.4	
Saif et al.	6.6			3		10.5
Belfiore et al.	14.9	11.4	8.8			
Ben-Porat et al.	40.4			27.7		
Al-Mulhim	11.6		5.4	7.1		
Zarshenas et al.	0		0	1	0	1

**Table 3:** Ferritin (%): Percentage (%) of patients with deficiencies at each postoperative visit.

Study	Preop (%)	3 Months (%)	6 Months (%)	12 Months (%)	24 Months (%)	36 Months (%)	60 Months (%)
Kehagias et al.	3.3					17.8	
Moize et al.	8.3		0	6.5	20.6		
Eltweri et al.				8			
Ben-Porat et al.	8.3			11.1			
Gillon et al.	3.3			11.6	20		36.2
Zarshenas et al.	8		11	15	18	24	

**Table 4:** Vitamin B12: Percentage (%) of patients with deficiencies at each postoperative visit.

Study	Preop (%)	3 Months (%)	6 Months (%)	12 Months (%)	24 Months (%)	36 Months (%)	60 Months (%)
Hakeam et al.	8.1		19.6	26.2			
Gehrer et al.	3	2	12	14	14		
Aarts et al.				9			
Ruiz-Tovar et al.	0	0	0	0	0		
Kehagias et al.	3.3						3.5
Damms-Machado et al.	9.3	4.8	9.8	17.2			
Moize et al.	2.7		3.7	3.2	5.9		12.5
Saif et al.				2.9			
Eltweri et al.				20			
Belfiore et al.	10.7	9	6				
Ben-Porat et al.	11.7			16.7			
Al-Mulhim	1.8		7.1	14.3			
Gillon et al.	6.5			19	12.8		3.8
Zarshenas et al.	1		3	0	0	0	

**Table 5:** Folate: Percentage (%) of patients with deficiencies at each postoperative visit.

Study	Preop (%)	3 Months (%)	6 Months (%)	12 Months (%)	24 Months (%)	36 Months (%)	60 Months (%)
Hakeam et al.	0		6.5	9.8			
Gehrer et al.	3	10	16		20		
Aarts et al.				15			
Ruiz-Tovar et al.	3.3	0	0	0	0		
Kehagias et al.	0					0	
Damms-Machado et al.	5.5	9.5	9.8	13.8			
Saif et al. ^1^				8.8	0	5.5	
Gjessing et al.	23			8			
Belfiore et al. ^1^	19.1	29.6	11.8				
Ben-Porat et al. ^1^	40.5			21.4			
Al-Mulhim	0.9			5.4	6.25		
Gillon et al.	8.8			12.3	7.6		10.6
Zarshenas et al.	0		0	0	0	0	

^1^ Additional iron supplementation for patients, further described in Appendix A.

**Table 6:** Vitamin D: Percentage (%) of patients with deficiencies at each postoperative visit.

Study	Preop (%)	3 Months (%)	6 Months (%)	12 Months (%)	24 Months (%)	36 Months (%)	60 Months (%)
Gehrer et al.	23		6	22	28		
Aarts et al.				39			
Ruiz-Tovar et al.	96.7	13.2	3.3	3.3	3.3		
Kehagias et al.							
Damms-Machado et al.	83	76.2	70.7	70.4			
Moize et al.	90		22.7	37	66.7		
Saif et al.	75			34		55	
Capoccia et al.							
Eltweri et al.				81			
Gjessing et al.	47			49			
Belfiore et al.	31.9	20.5	11.8				
Ben-Porat et al.	97.9			93.6			
Lanzarini al.	100			40.9	13.6		
Lanzarini al. ^1^	0			28.1	15		
Al-Mulhim	60		21	8.9			
Gillon et al.	20.4			4.9	8.2		6.7
Zarshenas et al.	46		25	14	19	20	

^1^ Vitamin D intervention group.

## Appendix A

**Table A1:** Additional information describing supplementation regimens for each study cited above.

Article #	Authors	Iron and Ferritin	B12	Folate	Vitamin D
**1**	Hakeam et al.	All patients who received omeprazole postop developed iron deficiency.	Patients with preop B12 deficiency given 1000 mcg intramuscular injection 1 day postop. 6 months postop, 6 patients received 1000 mcg IM for two months. 9 of 16 patients who developed B12 deficiency used omeprazole for at least 6 months postop.	0.2 mg folic acid, 12 mcg cyanocobalamin. No notes on compliance	
**2**	Gehrer et al.	100% of iron deficiency treated with IV injection of iron-III-hydroxide	Postop, 80% of B12 deficiency successfully treated with IM.		100% of Vitamin D deficiency treated with 300,000 IE of oily suspension cholecalciferol. Secondary hyperparathyroidism treated with normal Vitamin D supplementation.
**3**	Aarts et al.				Multivitamin 3× daily. No notes on compliance.
**4**	Ruiz-Tovar et al.				Multivitamin daily. No notes on compliance.
**5**	Kehagias et al.		B12 supplement IM 1000–3000 if deficient preop.		
**6**	Damms-Machad et al.		11% B12 injections		Vitamin D supplementation was protective for Vit D deficiency.
**7**	Moize et al.	160 mg of iron.	1000 mcg B12 IM monthly		880 IU Vitamin D.
**8**	Saif et al.	Iron + 1200 mg calcium citrate. No significant difference noted in Iron deficiencies based on supplementation compliance.			Vitamin D additionally prescribed based on need. No significant difference noted in Vitamin D deficiency based on supplementation compliance.
**9**	Capoccia et al.				
**10**	Eltweri et al.				Vitamin D daily. No notes on compliance.
**11**	Gjessing et al.				Vitamin D 10 mcg/day. No notes on compliance.
**12**	Belfiore et al.	Patients with iron deficiency given 329.7 mg ferrous sulfate.	Vitamin B12 deficiency treated orally or by 1000 mcg IM. Folate deficiency treated with 105 mg folate daily for 3 months.	Patients with folate deficiency given 20 mg folate.	
**13**	Ben-Porat et al.	Iron deficiency treated with 200 mg iron supplementation daily.		Folate deficiency treated with 105 mg folate daily for 3 months.	400 IU Vitamin D and 500 mg Calcium. No notes on compliance.
**14**	Lanzarini al.				16,000 IU calcifediol for patients with deficiency in intervention group. No notes on compliance. Intervention groups compared.
**15**	Al-Mulhim				Multivitamin daily. No notes on compliance.
**16**	Gillon et al.		B12 supplementation based on deficiency. Compliance well documented.	Folic acid supplementation based on deficiency. Compliance well documented.	Vitamin D supplementation based on deficiency. Compliance well documented.
**17**	Zarshenas et al.	Deficiencies corrected as needed.			1200 mg calcium daily. No notes on compliance. 50,000 IU prescribed for patients with preop Vitamin D deficiency. Other deficiencies corrected as needed.
